# Supplementary material for: WordCommentsAnalyzer: A windows software tool for qualitative research
Source: F1000Res. 2018 Sep 4;7:536. Originally published 2018 May 3. [Version 2] doi: 10.12688/f1000research.14819.2 (PMC6137411; doi:10.12688/f1000research.14819.2)
Supplement: Supplementary file 3 [file f1000research-7-17614-s0002.tgz › b2a79496-4ada-47c4-9682-a99ce867836d.zip!/Eight Iranian Health Journals/Journal of Knowledge and Health_7.docx]

© Journal of Knowledge and Health. This work is licensed under CC BY 4.0 (https://creativecommons.org/licenses/by/4.0/).

Volume & Issue: دوره 11، شماره 3: 1395

Title: تأثير الگوی توانمندسازی مراقبین خانگی بیماران نارسایی قلبی بر پذيرش مجدد بیمارستانی و مراجعه مجدد به پزشك در یک دوره 9 ماهه

http://knh.shmu.ac.ir/index.php/site/article/view/1258

مقدمه: توانمندکردن مراقبین خانگی یکی از اقدامات مؤثر در بهبود خودمراقبتی و کاهش پذيرش مجدد و مراجعه به پزشك در بیماران نارسایی قلبی است. هدف از اين پژوهش بررسي تأثير الگوی توانمندسازی مراقبین خانگی بیماران نارسایی قلبی بر پذيرش مجدد بیمارستانی و مراجعه مجدد به پزشك در یک دوره 9 ماهه بوده است. مواد و روش‌ها: پژوهش حاضر يك مطالعه كارآزمايي باليني تصادفي یک سو کور با حجم نمونه 97 نفر از بيماران مبتلا‌به نارسايي قلبي که با نمونه‌گيري به‌روش قابل دسترس انتخاب و سپس به‌صورت تصادفی ساده در دو گروه آزمون و شاهد قرار گرفتند. چك ليست نيازهاي مراقبتي بیماران نارسایی قلبی و مراقبین آنها در منزل مبنای مداخلات آموزشی و مراقبتی در این مطالعه بوده، مداخلات آموزشی و مراقبتی به‌صورت تئوری، عملی و ارايه فیلم و کتابچه آموزشی با بستری شدن بیمار شروع و سپس با ویزیت در منزل ادامه یافته است. تأثير مداخلات انجام شده بر پذيرش مجدد بیمارستانی و مراجعه مجدد به پزشك در دو گروه آزمون و شاهد مورد بررسي قرار گرفت. داده‌ها با استفاده نرم‌افزار SPSS وآزمون آماري تي مستقل، آزمون دقیق فیشر، آزمون خی دو و آمار توصيفي تجزيه و تحليل گردید. نتایج: ميانگين پذيرش مجدد در گروه آزمون 01/1±65/1 و در گروه شاهد 07/1±74/2 و ميانگين مراجعه به پزشك در گروه آزمون 24/1±73/2 و در گروه شاهد 94/±32/3 بوده و آزمون تي مستقل در میانگین پذیرش مجدد با 01/0=P و در میانگین مراجعه به پزشک با 009/0=P در دو گروه معني‌دار بوده است. نتیجه‌گیری: توجه به نیازهای مراقبین بیماران همراه با توانمندی آنان در فرآیند مراقبت ضمن اینکه ارايه مراقبت بهتر جهت بیماران را فراهم می‌کند، زمینه‌ساز کاهش پذیرش مجدد بیمارستانی و مراجعه آنان به مراکز درمانی خواهد بود.

Title: تأثیر خصوصیات اقتصادی- اجتماعی خانوارها و توسعه منطقه‌اي بر روی هزینه‌های بهداشتی و فاجعه‌بار سلامتی

http://knh.shmu.ac.ir/index.php/site/article/view/1320

مقدمه: باتوجه به حیاتی بودن نیازهای سلامت افراد، به هنگام بروز مشکلات سلامتی، نمی‌توان تقاضا برای خدمات درمانی جهت رفع مشکل سلامتی را، به تعویق انداخت. از سوی دیگر، بروز مشکلات سلامت همواره توأم با عوامل خطر زیادی بوده است و عموماً افراد قادر به پیش‌بینی زمان بروز مشکلات سلامت خود نیستند. لذا باید عوامل مؤثر بر روی این هزینه‌ها بررسی و تعیین‌کننده‌های هزینه‌های فاجعه‌بار سلامتی نیز مورد رصد قرار گیرد. مواد و روش‌ها: در این مطالعه با استفاده از آمار هزینه درآمد خانوارهای ایرانی در مناطق شهری و روستایی مربوط‌به سال 1391، با استفاده از یک الگوی رگرسیونی و یک الگوی probit و به کمک نرم‌افزار STATA به تجزيه و تحليل داده‌ها پرداخته شد. مطالعه حاضر برخی متغیرها، همچون سطح محرومیت استانی را نیز به مدل اضافه نموده است. ضرایب در سطوح معنی‌داری کمتر از 10% مورد ارزیابی قرار گرفته‌اند. نتايج: سهم هزینه‌های بهداشتی از بودجه خانوار و احتمال مواجهه با مخارج فاجعه‌بار سلامتی در خانوارهایی که در ادامه ذكر مي‌شود، بیشتر بوده است: در استان‌های توسعه‌یافته‌تر (مانند تهران) زندگی می‌کنند، سن سرپرست خانوار زیاد است، تعداد افراد بالای 60 سال و کمتر از 6 سال بیشتری دارند، در مناطق روستایی زندگی می‌کنند، مستأجر هستند و سطح تحصیلات سرپرست خانوار کمتر است، بیشتر بوده است.نتیجه‌گیری: این بررسی لزوم توجه به توزیع عادلانه امکانات بهداشتی در شهر و روستا، حمایت از اقشار آسیب‌پذیر مانند سالمندان، تأمین مسکن خانوارها، ارتقای سواد و آموزش عمومی، پوشش بیمه‌های همگانی و همچنین توسعه بیشتر مناطق محروم را مورد تأکید قرار می‌دهد. نتایج این مطالعه نشان داد كه سه گروه خانوارهای مستأجر، خانوارهای فقیر ساکن در کلان شهرها و سالمندان را می‌توان از آسیب‌پذیرترین اقشار جامعه دانست.

Title: کارآمدی آموزش جامع مهارت فرزندپروری سلامت محور به مادران بر اصلاح رفتار غذاخوردن کودکان دارای اضافه وزن

http://knh.shmu.ac.ir/index.php/site/article/view/1346

مقدمه: اضافه وزن و چاقی در کودکان با سرعت رو‌به افزایش است و پیش‌بینی می‌شود جمعیت کودکان چاق جهان تا سال 2020 به 1/9 درصد برسد؛ چاقی دوران کودکی با بسیاری از شرایط ناسازگار سلامت مانند بیماری عروق قلبی، دیابت نوع 2، افزایش چربی و فشارخون، ناخوشی و مرگ و میر در بزرگسالی ارتباط دارد. در همین راستا این پژوهش با هدف بررسی تأثیر آموزش جامع مهارت فرزندپروری سلامت محور به مادران بر اصلاح رفتار غذاخوردن کودکان انجام شده است. مواد و روش‌ها: در این مطالعه تعداد 50 نفر از مادران (به همراه فرزندشان) براساس معیارهای ورود- خروج با روش نمونه‌گیری در دسترس انتخاب و به تصادف در دو گروه مداخله و کنترل قرار گرفتند. گروه مداخله به‌صورت هفتگی و هر جلسه به مدت 90 دقیقه طی 14 جلسه تحت آموزش قرار گرفتند. هر یک از آزمودنی‌ها قبل و بعد از آموزش به کلیه گویه‌های پرسشنامه‌های دموگرافیک و رفتار غذاخوردن کودکان (CEBQ) پاسخ دادند. داده‌ها با استفاده از نسخه 21 نرم‌افزار آماری SPSS مورد تجزیه و تحلیل قرار گرفتند. نتایج: تحلیل یافته‌ها با روش تحلیل کوواریانس چندمتغیره نشان داد آموزش جامع مهارت فرزندپروری سلامت محور باعث اصلاح نمرات رفتار غذاخوردن کودکان (به جز میل به آشامیدنی) گروه مداخله در مقایسه با گروه کنترل شده است (05/0>P). نتیجه‌گیری: نتایج این بررسی نشان داد که آموزش جامع فرزندپروری سلامت محور به مادران، به اصلاح رفتار غذاخوردن کودکان دارای اضافه وزن منتهی می‌شود. همچنین به‌نظر می‌رسد که شاخص توده‌بدنی (BMI) کودکان به‌واسطه اصلاح رفتار غذا خوردن، کاهش خواهد یافت.

Title: پیش‌بینی‌کننده‌های خستگی در بیماران مبتلابه بیماری انسدادی مزمن ریه

http://knh.shmu.ac.ir/index.php/site/article/view/1348

مقدمه: خستگي يكي از علایم شایع در بیماران مبتلا‌به بیماری انسدادی مزمن ریه (COPD: Chronic obstructive pulmonary disease) می‌باشد كه مي‌تواند ابعاد مختلف زندگي آنها را تحت‌الشعاع قرارداده و مشكلات عديده‌اي را برای آنان به‌وجود آورد. این مطالعه با هدف تعیین پیش‌بینی‌کننده‌های خستگی در بیماران مبتلا‌به بیماری انسدادی مزمن ریه انجام شد. مواد و روش‌ها: پژوهش حاضر یک مطالعه توصیفی- تحلیلی بوده که برای بیماران مبتلا‌به بیماری انسدادی مزمن ریه بستری در بخش فوق تخصصی ریه بیمارستان رازی رشت در سال 1394 انجام گرفت. در این پژوهش نمونه‌گیری به‌صورت در دسترس بوده و تعداد 70 بیمار مبتلا‌به بیماری انسدادی مزمن ریه از میان جامعه پژوهش انتخاب گردیدند. پرسشنامه اطلاعات دموگرافیک و مرتبط با بیماری و همچنین پرسش‌نامه چند بعدی خستگی (شامل خستگی عمومی، خستگی جسمی، خستگی ذهنی، کاهش فعالیت، کاهش انگیزه) براساس گفته‌های بیماران و پرونده آنان تکمیل گردید. برای تجزیه و تحلیل داده‌ها از نرم‌افزار SPSS نسخه 21 و از آمار توصیفی و آزمون‌های آماری استنباطی استفاده گردید. نتایج: میانگین سنی بیماران 07/11±5/70 سال می‌باشد. اکثریت واحدهای مورد پژوهش را مرد، بی‌سواد و کشاورز تشکیل داده‌اند. بیشترین میانگین نمره خستگی مربوط‌به بعد خستگی جسمی و کمترین میانگین نمره مربوط‌به بعد خستگی ذهنی است. با استفاده از مدل رگرسیون چند متغیره، سن (001/0=P) و بیماری قلبی (042/0=P) به‌عنوان پیش‌بینی‌کننده نمره خستگی در کل محسوب شده و این دو متغیر 49 درصد تغییرات در نمره خستگی را پیش‌بینی می‌کردند (49/0=R2). سن و بیماری قلبی پیش‌بینی‌کننده‌های خستگی عمومی با (39/0=R2) و سن از پیش‌بینی‌کننده‌های خستگی جسمی (27/0=R2)، کاهش فعالیت (29/0=R2) و کاهش انگیزه (32/0=R2)، بوده است. پیش‌بینی‌کننده‌های خستگی ذهنی هم شامل بیماری قلبی و تعداد دفعات بستری فرد در بیمارستان به‌علت بیماری COPD در طی یک سال گذشته می‌باشد (41/0=R2). نتیجه‌گیری: در کنار ارايه مراقبت‌های جسمی و مداخلات پرستاری، در نظر گرفتن عوامل دموگرافیک، بیماری‌های زمینه‌ای و ارایه مداخلات روانشناختی براساس خستگی بیماران در مدیریت همه جانبه مشکلات بیماران COPD، علی‌الخصوص خستگی این بیماران، از اهمیت به‌سزایی برخوردار می‌باشد.

Title: مدل‌سازی تصفیه فاضلاب لبنی به‌روش انعقاد الکتریکی با استفاده از سامانه استنتاج فازي– عصبي تطبيقي

http://knh.shmu.ac.ir/index.php/site/article/view/1398

مقدمه: پیش‌بینی شاخص‌های کیفی تصفیه فاضلاب دارای اهمیت زیادی در روش‌های تصفیه فاضلاب مدرن است. یکی از مهمترین مشکلات در هنگام پیش‌بینی کارآیی فاضلاب تصفیه شده، پیچیدگی ویژگی‌های فیزیک و شیمیایی فاضلاب اولیه و اختلاف داده‌ها به علل مختلف است. مدل‌سازي تصفیه فاضلاب با استفاده از سامانه استنتاج فازي- عصبي تطبيقي مي‌تواند به بهبود فرآيند كنترل كيفيت فاضلاب كمك كند. هدف از اين مطالعه، مدل‌سازی تصفیه فاضلاب لبنی به‌روش انعقاد الکتریکی با استفاده از سامانه استنتاج فازي- عصبي تطبيقي است. مواد و روش‌ها: در این مطالعه از روش سامانه استنتاج فازي- عصبي تطبيقي برای پیش‌بینی فرآیند تصفیه فاضلاب به‌روش انعقاد الکتریکی استفاده شد. شاخص‌های زمان، ولتاژ، TSS (Total suspended solids) و BOD (Biochemical oxygen demand) به‌عنوان ورودی و راندمان حذف COD (Chemical oxygen demand) به‌عنوان خروجی در نظر گرفته شد. همچنین برای بهینه‌سازی مدل از توابع عضویت مختلف، تعداد توابع عضویت و دوره‌‌های یادگیری متعددی به شکل آزمون و خطا استفاده شد. نتایج: بهترين مدل با استفاده از تابع عضویت زنگوله‌ایی، تعداد توابع 3 3 3 3 3 و چرخه يادگيري 300 به‌دست آمد كه دارای كمترين ميانگين مربعات خطا و بهترين ضريب تبيين (R2) بود. مقدار ضريب تبيين بهترین مدل و ميانگين مربعات خطا به‌ترتیب 9912/0 و 012/0 بود. نتیجه‌گیری: تجزیه و تحلیل مدل نشان داد که سامانه استنتاج فازي- عصبي تطبيقي یک ابزار قدرتمند برای پیش‌بینی کارآیی تصفیه فاضلاب لبنی به‌روش انعقاد الکتریکی است.

Title: تأثیر یک دوره تمرینات هوازی بر بیان ژن MTNR1B و سطوح انسولین و گلوکز در پانکراس رت‌های دیابتی شده با استرپتوزوتوسین-نیکوتین آمید

http://knh.shmu.ac.ir/index.php/site/article/view/1416

مقدمه: دیابت نوع 2 بیماری مزمنی است که با سرعت هشداردهنده‌ای در جهان در حال گسترش است. اگرچه دیابت نوع 2 یک بیماری چند علتی است، ولی اخیراً مشخص شده است که ژن MTNR1B با میزان ابتلا به دیابت نوع2 مرتبط است و افزایش بیان آن خطر دیابت نوع 2 را افزایش می‌دهد. هدف از این مطالعه بررسی تأثیر 12 هفته تمرینات هوازی بر بیان ژن MTNR1B و گلوکز و انسولین ناشتا در رت‌های نر ویستار مبتلا‌به دیابت نوع 2 است. مواد و روش‌ها: پژوهش حاضر از نوع بنیادی و روش آن تجربی می‌باشد. در این مطالعه از 30 سر رت نر نژاد ویستار با محدوده وزنی 20±220گرم، استفاده شد. در طول دوره رت‌ها در شرایط استاندارد یعنی دمای 3±22سانتی‌گراد و رطوبت 45 درصد و دوره‌های 12 ساعته متوالی نور و تاریکی نگهداری شدند. رت‌ها به سه گروه  تقسیم شدند. القای دیابت از طریق محلول نیکوتین آمید و STZ انجام شد. برنامه تمرینی برای مدت 12 هفته به تعداد 5 جلسه در هفته با افزایش تدریجی سرعت (18 الی 26 متر بر دقیقه) و زمان (10 الی 55 دقیقه) در قالب دویدن روی تریدمیل انجام گرفت. غلظت گلوکز به‌روش آنزیمی رنگ سنجی گلوکز اکسیداز، انسولین به‌روش الیزا و بیان ژن به‌روش RT-Real time PCR اندازه‌گیری شد. کليه بررسي‌هاي آماري با استفاده از نرم‌افزار SPSS/Win نسخه 16 انجام شد. نتایج: برنامه تمرینی به کاهش معنی‌دار سطوح گلوکز ناشتا در گروه دیابتی هوازی نسبت‌به گروه دیابتی کنترل منجر شد (001/0=P). سطوح انسولین سرم در گروه دیابتی هوازی بالاتر از گروه دیابتی کنترل بود اما این تفاوت به لحاظ آماری معنی‌دار نبود (148/0=P) از طرفی، اجرای تمرینات هوازی بیان ژن MTNR1B در بافت پانکراس را به میزان 39 درصد نسبت‌به گروه کنترل دیابتی کاهش داد (016/0=P). نتیجه‌گیری: نتایج این مطالعه نشان دادکه دوازده هفته ورزش هوازی به کاهش معنی‌دار گلوکز خون و کاهش معنی‌دار بیان ژن MTNR1B در بافت پانکراس نسبت‌به گروه کنترل دیابتی منجر شد.

Title: بررسي ميزان آلودگي انگلی كبد شترهای كشتار شده در شاهرود در سال 1394

http://knh.shmu.ac.ir/index.php/site/article/view/1429

مقدمه: با‌توجه به شرايط آب و هوايي ايران، شتر مي‌تواند به‌عنوان منبع پروتئيني ارزشمندي در آب و هواي كويري در نظر گرفته شود. آلودگي‌هاي انگلي از شايع‌ترين امراض دامي بوده كه علاوه‌بر زيان‌ها و مخاطرات بهداشتي، خسارات اقتصادي فراواني را به‌دنبال دارد. اجراي اين پروژه با هدف شناسايي انگل‌هاي شايع در كبد شتر صورت گرفته تا با كمك به اجراي برنامه مبارزه عليه بيماري‌هاي انگلي بتوان اقدام مؤثری در كاهش اين‌گونه بيماري‌ها نمود. مواد و روش‌ها: اين پروژه از فروردين سال 94 تا پایان مهرماه 94 صورت گرفته است، جهت تخمين ميزان آلودگي انگلي كبدهاي شتر، تعداد 94 نمونه از کبد شترهای کشتار شده در کشتارگاه شاهرود گرفته و نمونه‌ها پس از برش و خرد کردن به‌صورت قطعات دو تا سه سانتی‌متري جمع‌آوری و پس از شناورسازی و الک کردن، میزان آلودگی آن محاسبه گردید. نتايج: شايع‌ترين علت ضبط كبد در تعداد 94 نفر شتر كشتار شده، كيست هيداتيك مي‌باشد كه با 45/7 درصد بيشترين آلودگي را به خود اختصاص داده است. در رتبه بعدي، فاسيولا با فراواني 06/1 درصد قرار داشت اما هيچ‌گونه آلودگي انگلي ديكروسلیوم مشاهده نگرديد. ضمناً باتوجه به بررسي صورت گرفته، صددرصد شتراني كه به كيست كبدي آلوده بودند داراي آلودگي انگلي ريوي نيز بودند. نتیجه‌گیری: اين مطالعه نشان مي‌دهد كه شيوع آلودگي کبدهای شتر کشتار شده در شاهرود به انگل‌هاي فوق قابل توجه مي‌باشد كه اين مسأله علاوه‌بر تحميل زيان‌هاي اقتصادي ناشي از ضبط اندام آلوده، نشان‌دهنده وجود زمينه خطرات بهداشتي براي مصرف‌کنندگان منطقه مي‌باشد. لذا با‌توجه به چرخه زندگي و انتقال اين انگل‌ها، به‌كارگيري روش‌هاي پيشگيري بيماري با كمك سازمان دامپزشكي و اقدامات بهداشتي و كنترلي مناسب را طلب مي‌نمايد. همچنین با‌توجه به مخاطرات بهداشتي آلودگي انگلي و آلودگي قابل توجه شترها به انگل‌هاي بررسي شده، به‌كارگيري روش‌هاي پيشگيري بيماري با كمك شترداران و سازمان دامپزشكي ضروري مي‌باشد.

Title: استفاده از لام کوت شده تیموس گوساله جهت تشخیص آنتی‌بادی ضدهسته در سرم بیماران مبتلا‌به بیماری لوپوس

http://knh.shmu.ac.ir/index.php/site/article/view/1458

مقدمه: آزمون آنتی‌بادی ضدهسته‌ای رایج‌ترین روش جهت شناسایی بیماران مبتلا‌به لوپوس می‌باشد که متأسفانه به‌دلیل انحصاری بودن آن واردات آن به کشور سخت و هزینه‌بر است. هدف از مطالعه حاضر استفاده از لام کوت شده تیموس گوساله جهت تشخیص لوپوس بود. مواد و روش‌ها: تعداد 48 نمونه سرم (25 منفی و 23 مثبت) اخذ شده از بیماران مظنون به لوپوس جمع‌آوری شد. نمونه تیموس گوساله از کشتارگاه تهیه شد و با استفاده از میکروتوم برش‌هایی به ضخامت 5 میکرومتری از تیموس تهیه شدند. همچنین لام آماده کبد رت خریداری شد و به موازات آن آزمون استاندارد HEp-2 برای هر نمونه آزمایش گردیدند. جهت تعیین تیتر آنتي‌بادي پنج رقت دو برابر شونده‌ی از سرم (1:40 تا 1:640) با استفاده از بافر فسفات تهیه شده و آزمایش شدند. لام‌های رنگ شده با میکروسکوپ فلورسانس، با بزرگنمایی 200 میکروسکوپ بازبینی شدند و وجود فلورسانس سبز درخشان در هسته‌ی سلول‌ها به‌عنوان نتیجه مثبت تلقی شد. نتایج: نتایج این تحقیق نشان داد که استفاده از سلول‌های تیموس گوساله با حساسیت، ویژگی و دقتی برابر با 6/82% ، 100% و 7/91% نسبت‌به آزمون استاندارد در تشخیص آنتی‌بادی‌های ضدهسته‌ای (ANA) مفید بود. ارزش پیشگویی منفی و ارزش پیشگویی مثبت لام‌های تیموسی برابر 2/86% و 100 % به‌دست آمد. نتيجه‌گيري: به‌عنوان یک آزمون غربالگرانه‌ی ارزان و در دسترس، می‌توان از سلول‌های تیموس گوساله برای شناسایی ANA در سرم بیماران SLE لوپوس اریتماتو سیستمیک استفاده کرد. اگرچه بهینه‌سازی شرایط تهیه و آزمایش و تعیین نقطه Cut off مناسب می‌تواند کارایی آن را به 100% برساند.

Title: ارزیابی تأثیر امواج 2100 مگاهرتز گسیل شده از نسل سوم تلفن‌های همراه بر ایجاد استرس اکسیداتیو و جذب ریوی 99mTC-MIBI در ریه موش صحرایی نر

http://knh.shmu.ac.ir/index.php/site/article/view/1480

مقدمه: رادیوداروی 99mTC-MIBI در اسکن‌های قلبی به‌طور اختصاصی در قلب تجمع می‌یابد ولی جذب قابل توجهی نیز در ریه دارد. افزایش جذب ریوی ممکن است باعث تغییر جذب قلبی شود و با‌توجه به مجاورت قلب با ریه در کیفیت تصویر اسکن شده مؤثر است. بنابراین هدف از این مطالعه بررسی میزان تجمع ریوی رادیوداروی 99mTC-MIBI در اثر تابش امواج 2100 مگاهرتز تلفن همراه می‌باشد. مواد و روش‌ها: در این مطالعه موش‌های صحرایی نر نژاد ویستار پس از تزریق وریدی رادیوداروی 99mTC-MIBI، در قفس قرار گرفته و گروه‌های آزمون به‌وسیله گوشی تعبیه شده در زیر قفس، به‌مدت (15 و 30) دقیقه تحت تابش امواج 2100 مگاهرتز قرار گرفتند. برای گروه‌های شاهد تلفن همراه در حالت Standby قرار داشت. اکتیویته ریه توسط دزکالیبراتور و وزن ریه به‌وسیله ترازوی دیجیتال اندازه‌گیری شد و مقادیر سرمی MDA و Catalase با تکنیک الایزا مورد سنجش قرار گرفت. نتایج: تابش امواج 2100 مگاهرتز به مدت 15 و 30 دقیقه، به‌طور معنی‌داری جذب ریوی 99mTC-MIBI را افزایش داده و باعث ایجاد روند کاهشی معنی‌دار تغییرات اکتيویته ویژه ریه با زمان می‌شود ولی هیچ تغییر قابل توجهی در سطح استرس اکسیدایتو ایجاد نمی‌کند. نتیجه‌گیری: چنانچه فردی به‌منظور تهیه اسکن قلب مورد تزریق رادیوداروی 99mTC-MIBI قرار گیرد و در فاصله زمانی تزریق تا تهیه اسکن تحت تابش امواج 2100 مگاهرتز تلفن همراه قرار داشته باشد به‌علت افزایش جذب رادیودارو در ریه ممکن است اسکن قلبی دچار آرتیفکت شود و تشخیص بیماری به درستی صورت نگیرد.

Title: استفاده از تکنیک‌های هوش مصنوعی برای شناسایی میزان شدت بیماری کبد چرب غیر الکلی توسط شاخص‌هاي بالینی

http://knh.shmu.ac.ir/index.php/site/article/view/1369

مقدمه: بیماری کبد چرب غیر الکلی ( Non-alcoholic fatty liver disease:NAFLD) یکی از شایع‌ترین بیماری‌های کبدی است که شدت آن دارای سطوح مختلفی می‌باشد. اخیراً، دستگاه فیبرواسکن به‌عنوان یک روش غیر‌تهاجمی برای اندازه‌گیری میزان ارتجاع‌پذیری کبد و درنتیجه چرب بودن آن مورد استفاده قرار می‌گیرد. هدف از این پژوهش، ارايه یک روش کم هزینه‌ و ساده برای تشخیص این بیماری از طریق علايم بالینی می‌باشد. مواد و روش‌ها: در این تحقیق از یک مجموعه داده شامل 726 بیمار استفاده شد که هر یک دارای عارضه کبد چرب با شدت مختلفی بودند. برای هر بیمار، شدت بیماری توسط دستگاه فیبرواسکن اندازه‌گیری و آزمایشات بالینی و سنوگرافی نیز انجام گرفت. سپس به‌منظور تعیین رابطه بین اطلاعات به‌دست آمده از بیماران و سطوح بیماری از شبکه‌های عصبی مصنوعی استفاده شده است. در نهایت، به کمک تکنیک‌های هوش مصنوعی روشی برای استخراج قانون از شبکه‌های عصبی مصنوعی برای نمایش ارتباط بین داده‌ها استفاده شده است. نتایج: براساس نتایج به‌دست آمده از دستگاه فیبرواسکن، از بین 726 بیمار موجود، 5 مورد در کلاس F4، 23 مورد در کلاس F3، 132 مورد در کلاس F2، 151 مورد در کلاس F1 و 415 مورد در کلاس F0 (افراد سالم) قرار می‌گیرند. طبق روش پیشنهادی، دقت در شناسایی نمونه‌های هر کلاس به‌ترتیب: 100% برای کلاس F4، 99/31% برای کلاس F3، 93/94% برای کلاس F2 و 58/80% برای کلاس F1 می‌باشد. بر این اساس این روش می‌تواند نمونه‌های دسته‌های F4 و F3 را به‌صورت ایده‌آل و نمونه‌های دسته‌های F2 و F1 را با دقت خوبی شناسایی کند. نتيجه‌گيري: نتایج حاصل از این تحقیق نشان می‌دهد که روش پیشنهادی قادر است علاوه‌بر صرف هزینه کمتر و قابلیت دسترسی آسان‌تر در شناسایی بیماری کبد چرب غیر الکلی (NAFLD)، نحوه تشخیص بیماری و شرایط هر سطح از بیماری را در قالب مجموعه قوانینی برای تشخیص بیماری (بدون ادامه نیاز به شبکه عصبی) برای پزشکان تعیین نماید.
